# Supplementary figures and images for: YAP/TAZ enhances P-body formation to promote tumorigenesis
Source: eLife. 2024 Jul 24;12:RP88573. doi: 10.7554/eLife.88573 (PMC11268890; doi:10.7554/eLife.88573)

Figure 1-Figure supplement 1C

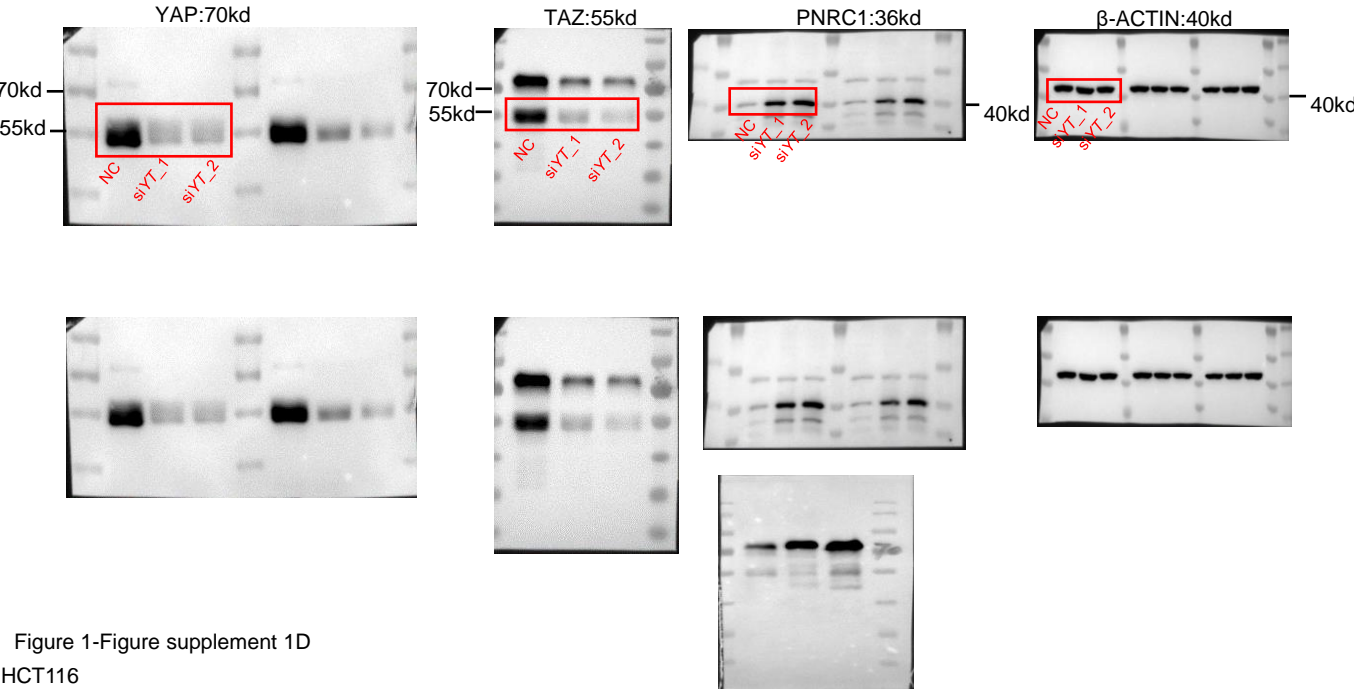

Figure 1-Figure supplement 1D  
HCT116

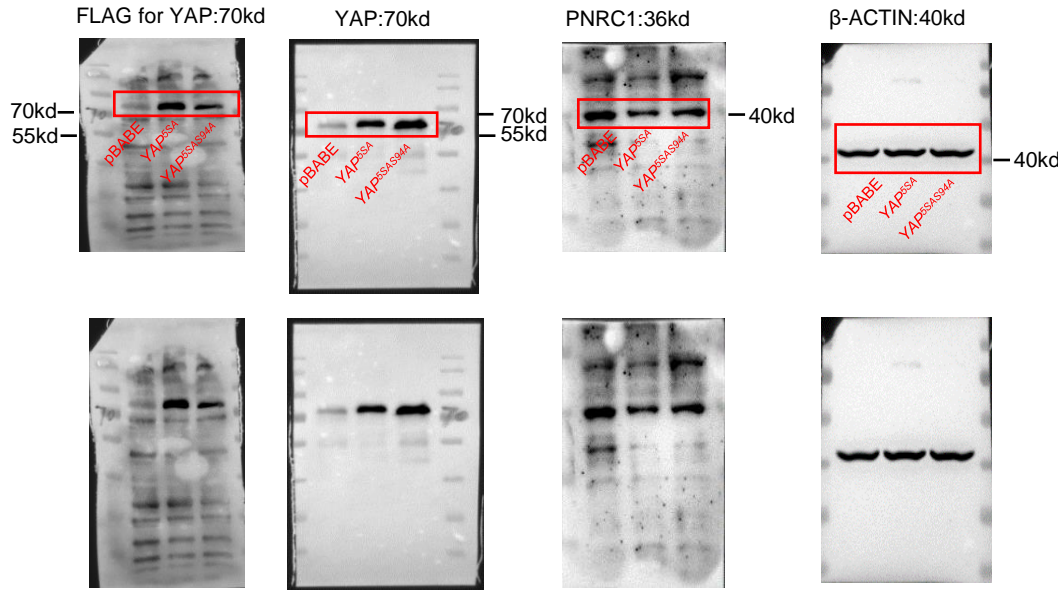

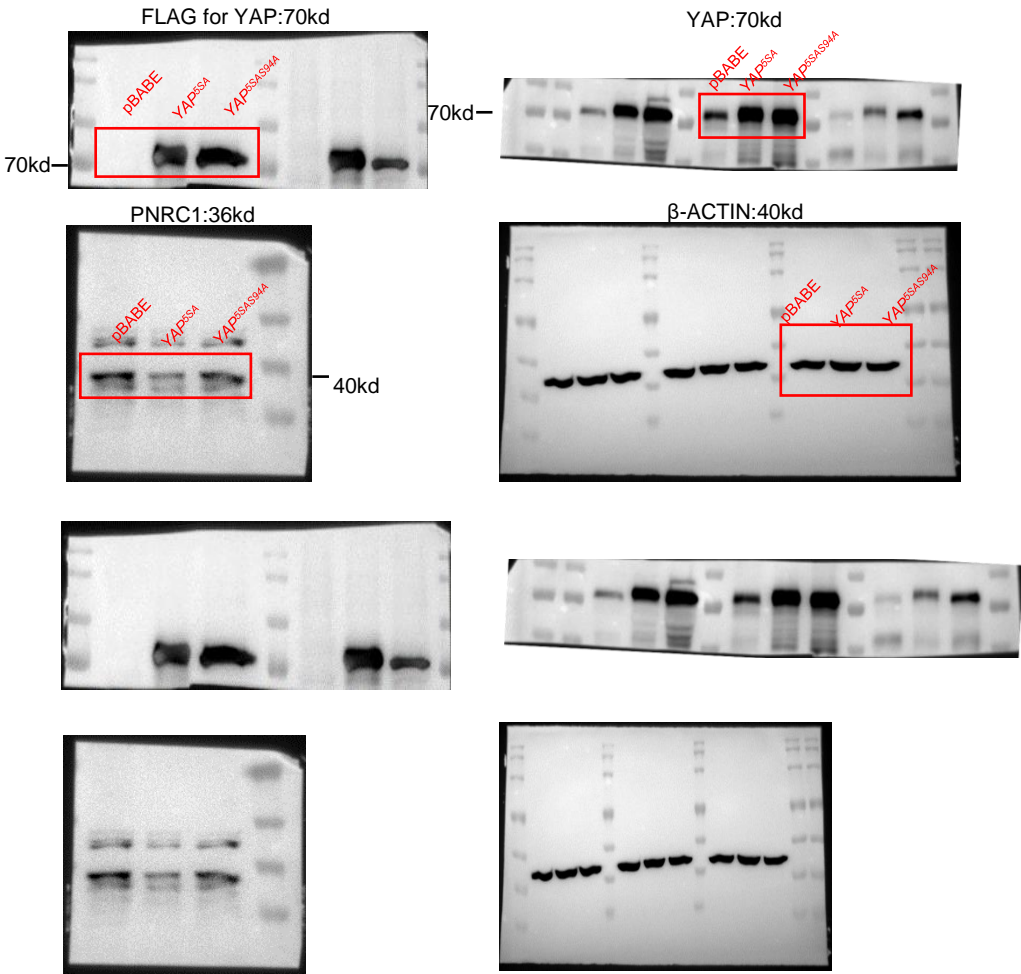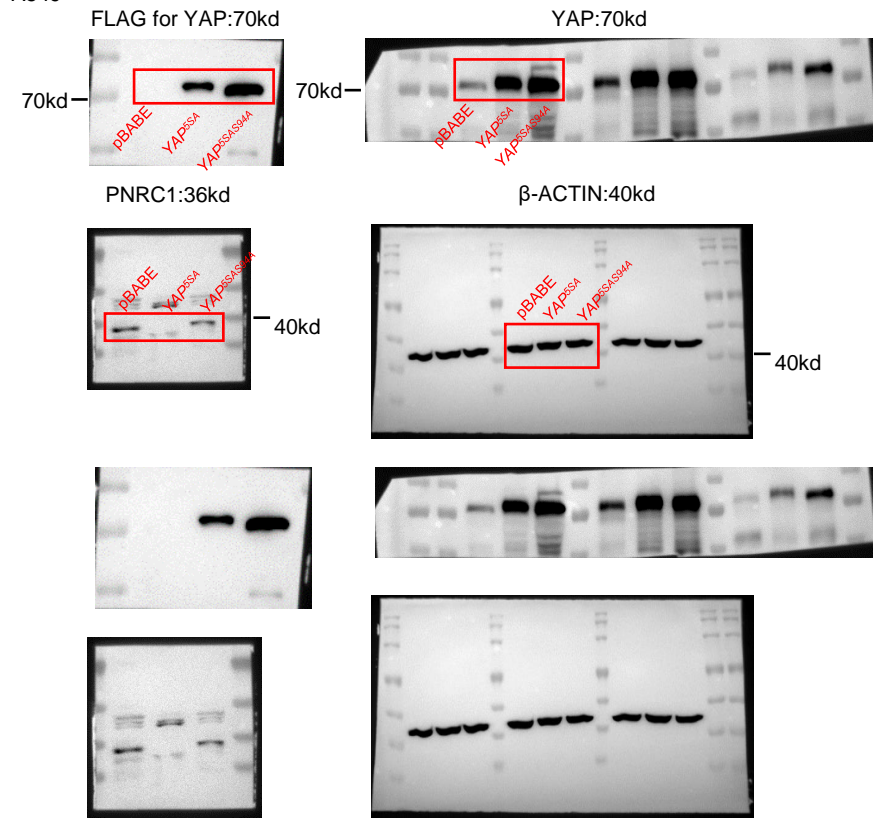

Supplement: Figure 1—figure supplement 1—source data 2. [file elife-88573-fig1-figsupp1-data2.pdf]

Figure 3-Figure supplement 1D

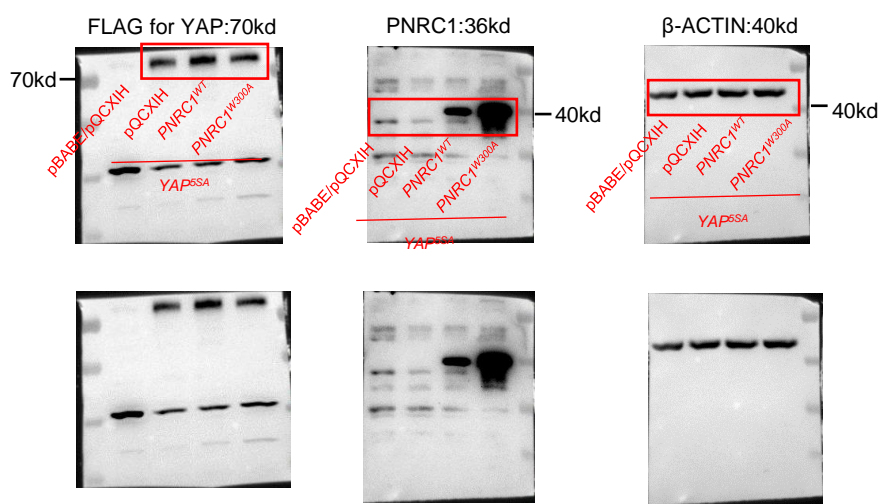

Supplement: Figure 3—figure supplement 1—source data 2. [file elife-88573-fig3-figsupp1-data2.pdf]

Figure 4-Figure supplement 1A

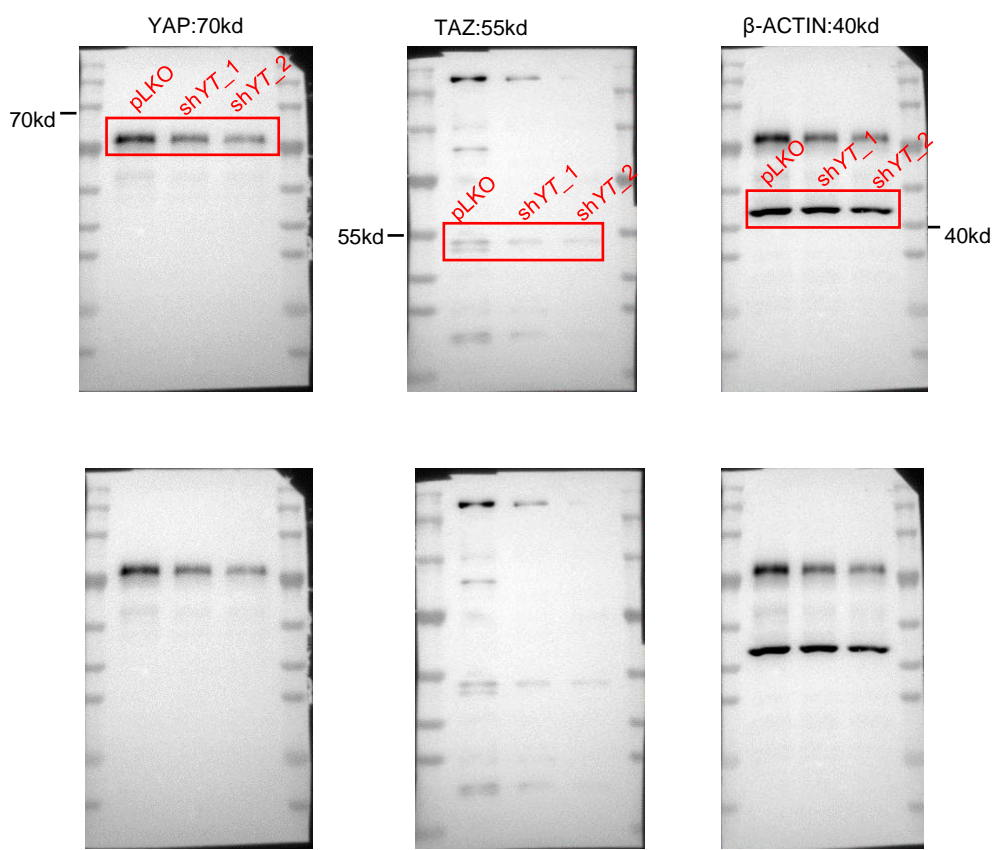

Supplement: Figure 4—figure supplement 1—source data 2. [file elife-88573-fig4-figsupp1-data2.pdf]

Figure 6-Figure supplement 1A

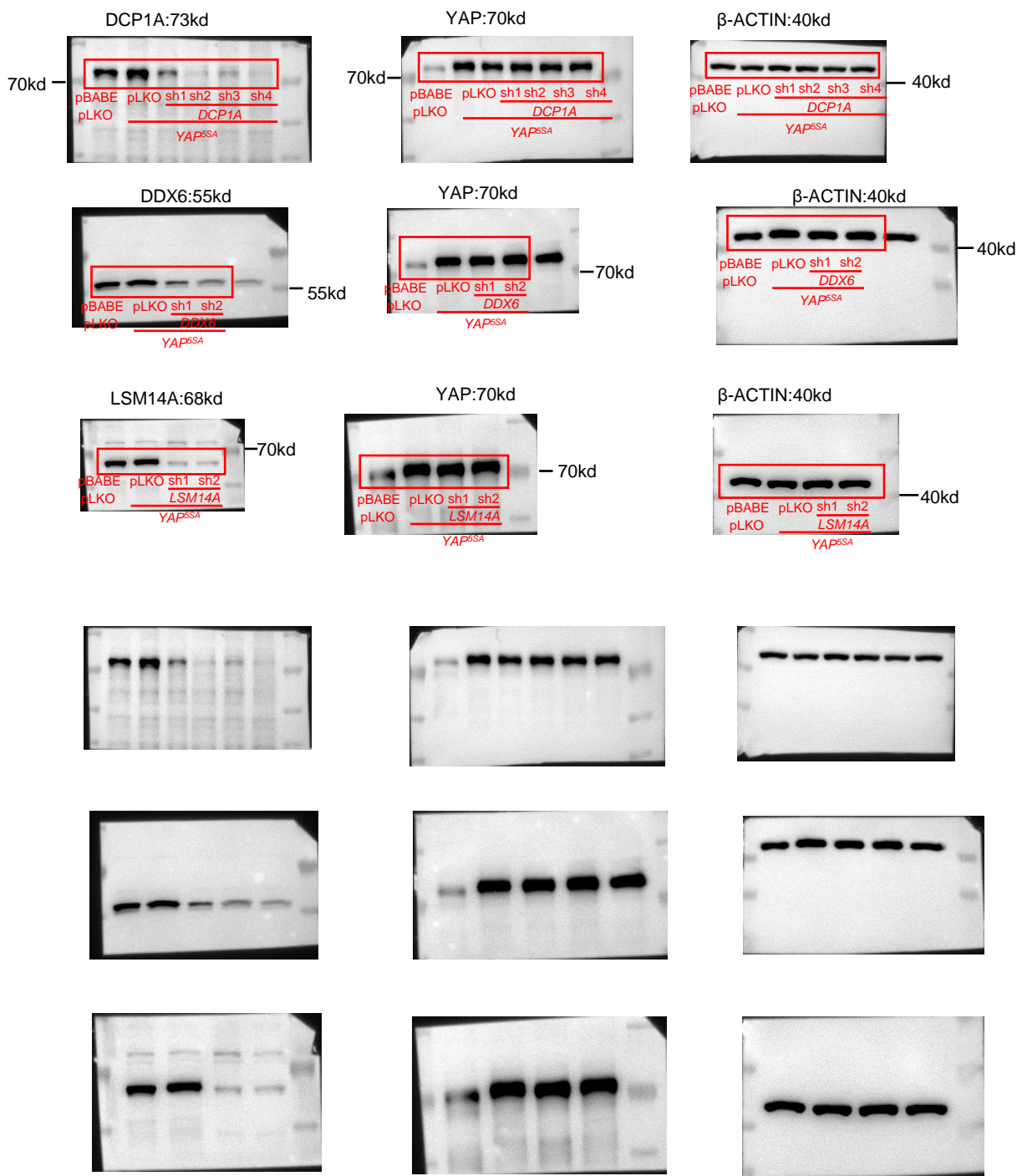

Supplement: Figure 6—figure supplement 1—source data 2. [file elife-88573-fig6-figsupp1-data2.pdf]

Figure 6-Figure supplement 2A

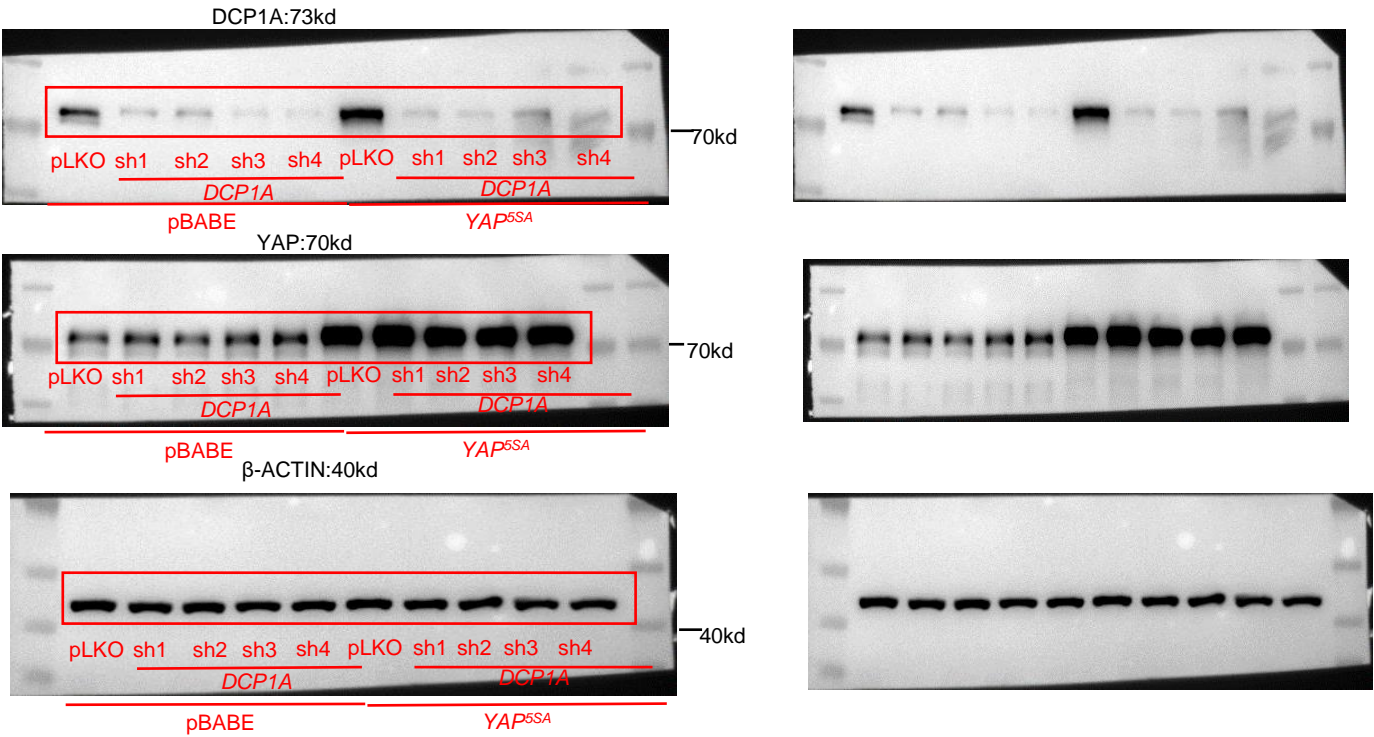

Figure 6-Figure supplement 2B

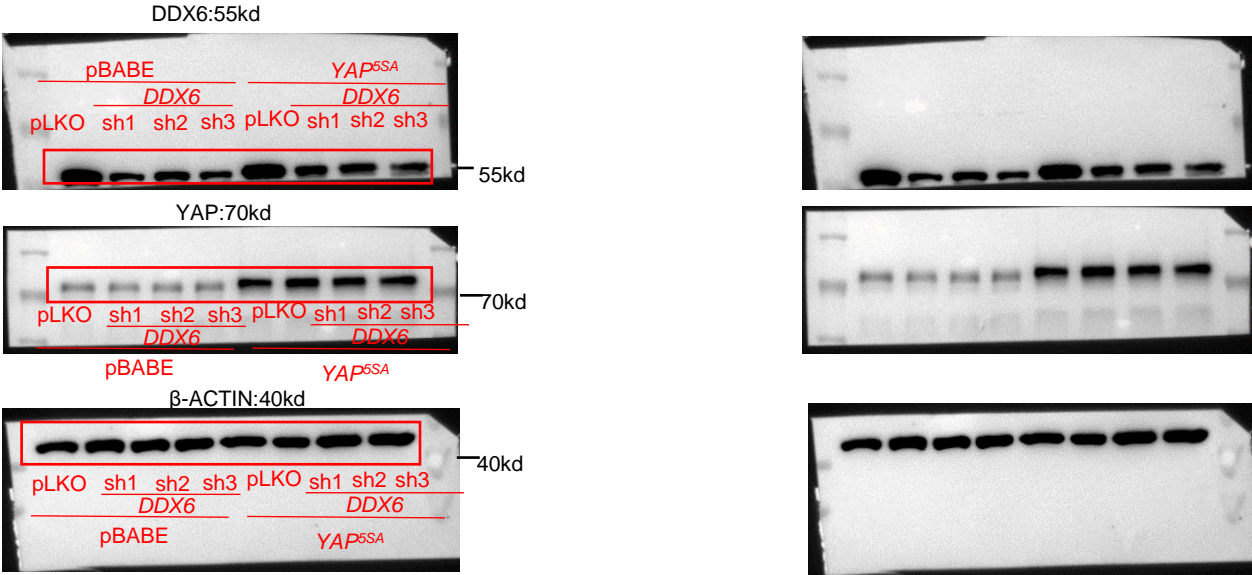

Figure 6-Figure supplement 2C

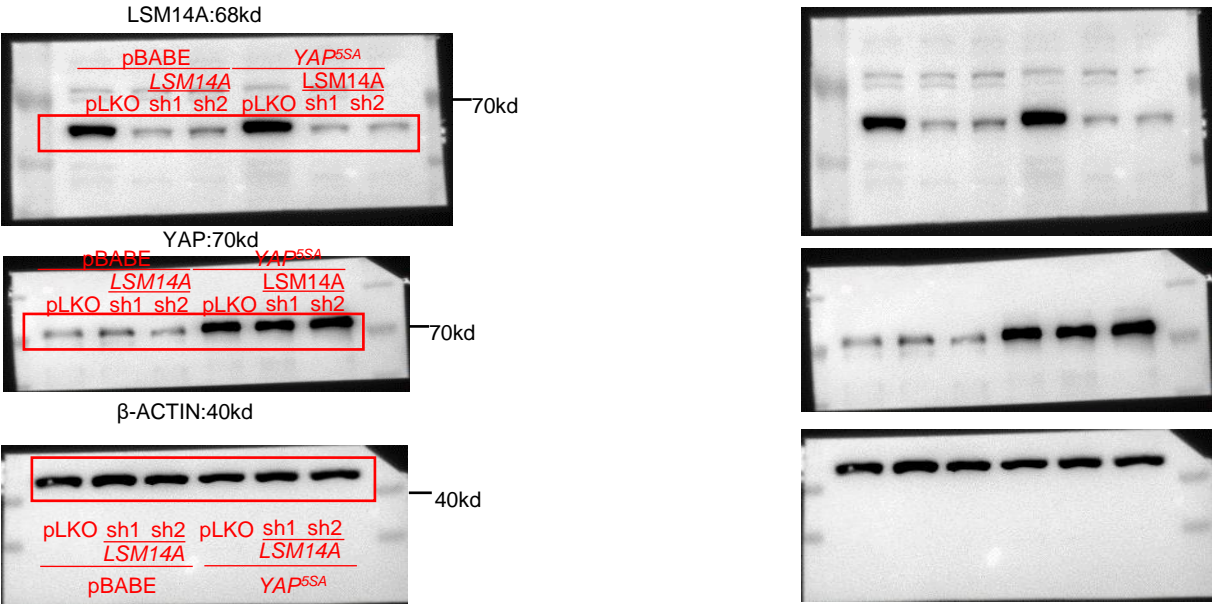

Supplement: Figure 6—figure supplement 2—source data 2. [file elife-88573-fig6-figsupp2-data2.pdf]

Figure 6-Figure supplement 4C

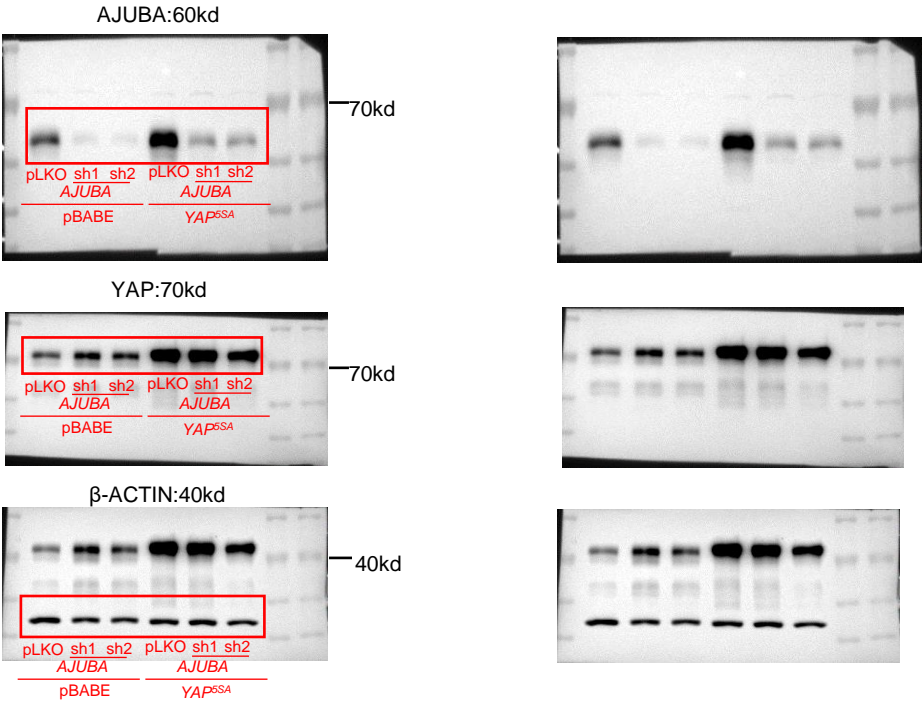

Supplement: Figure 6—figure supplement 4—source data 2. [file elife-88573-fig6-figsupp4-data2.pdf]

Figure 7-Figure supplement 3A

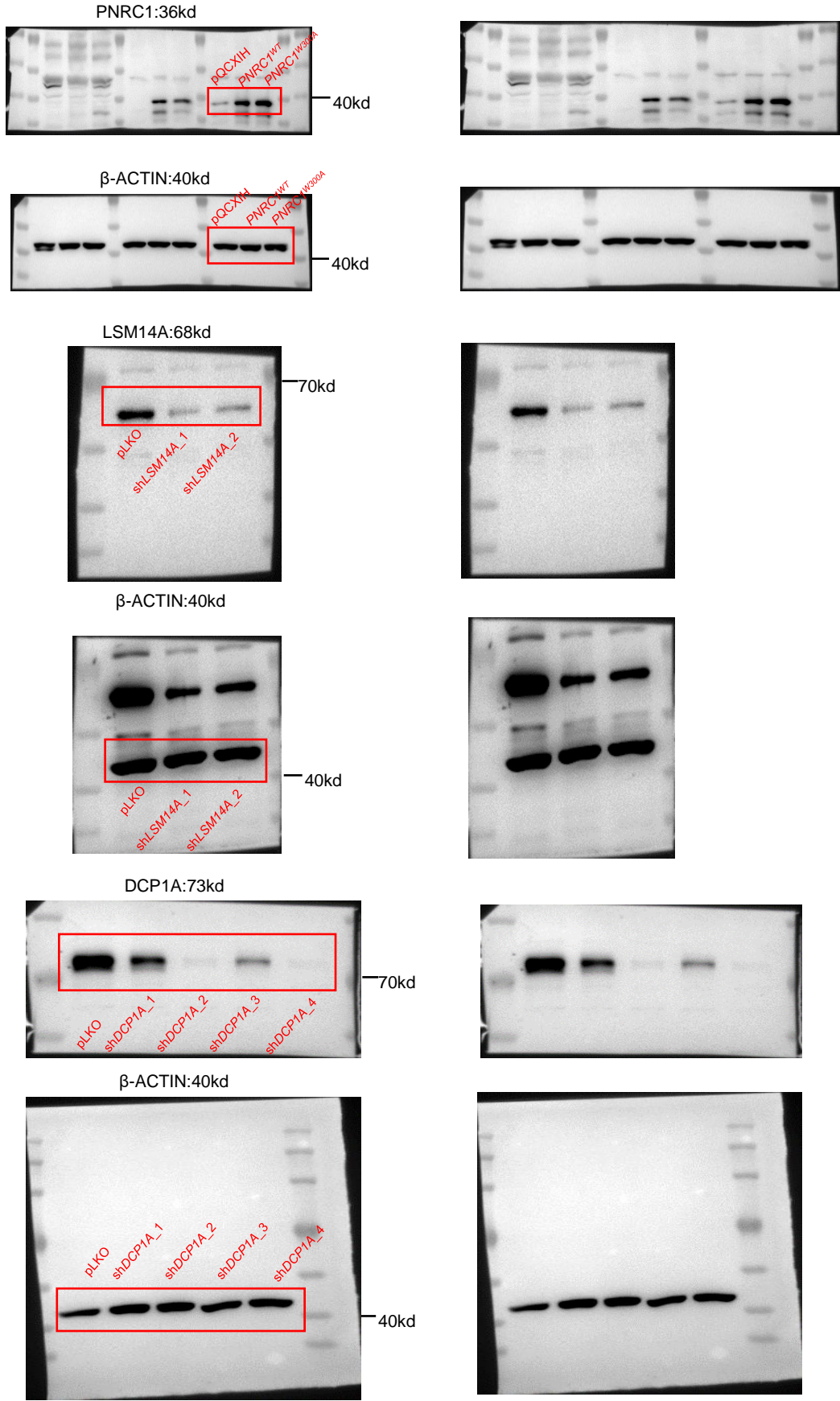

Supplement: Figure 7—figure supplement 3—source data 2. [file elife-88573-fig7-figsupp3-data2.pdf]

Figure 7-Figure supplement 4A

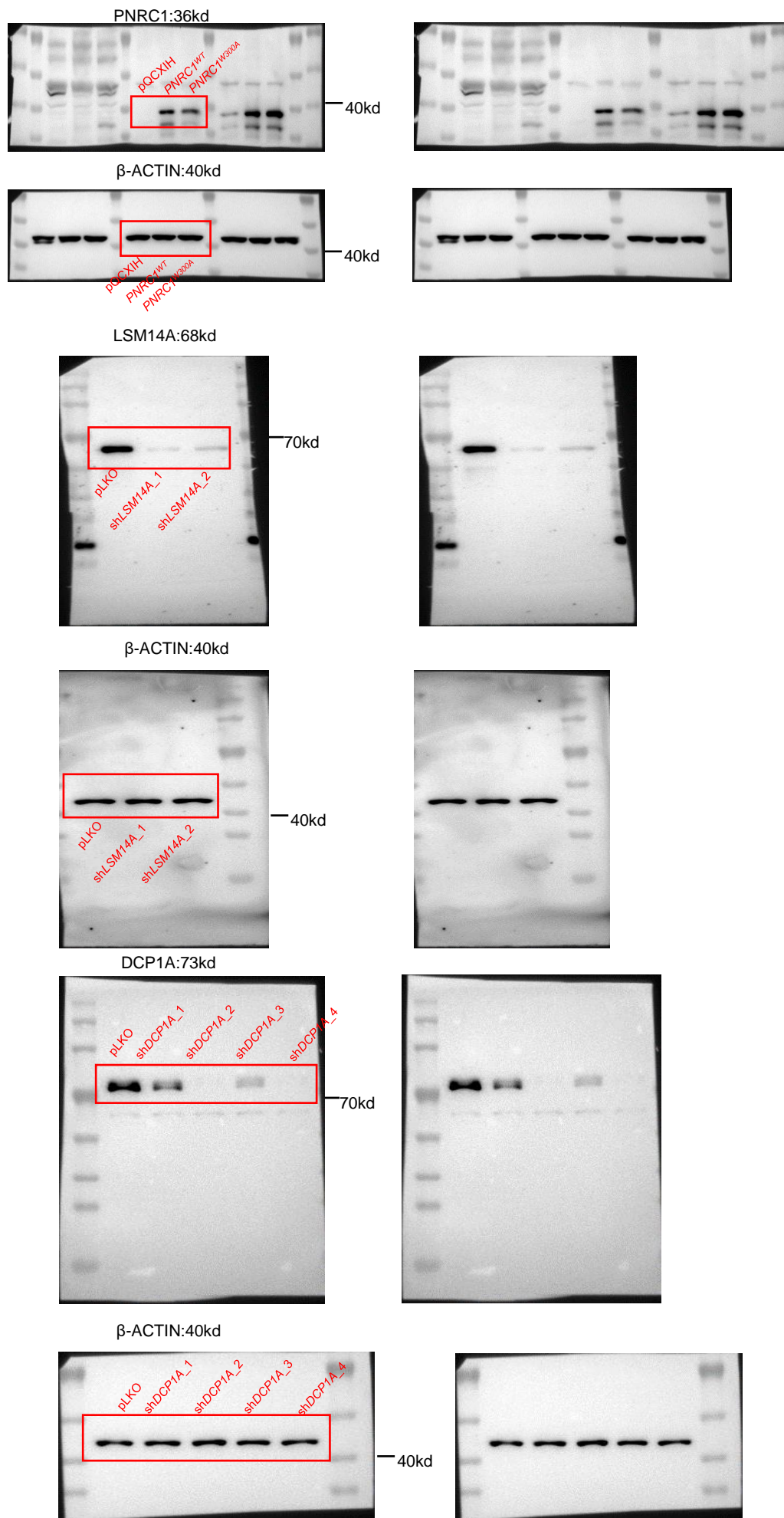

Supplement: Figure 7—figure supplement 4—source data 2. [file elife-88573-fig7-figsupp4-data2.pdf]

Figure 7-Figure supplement 5A

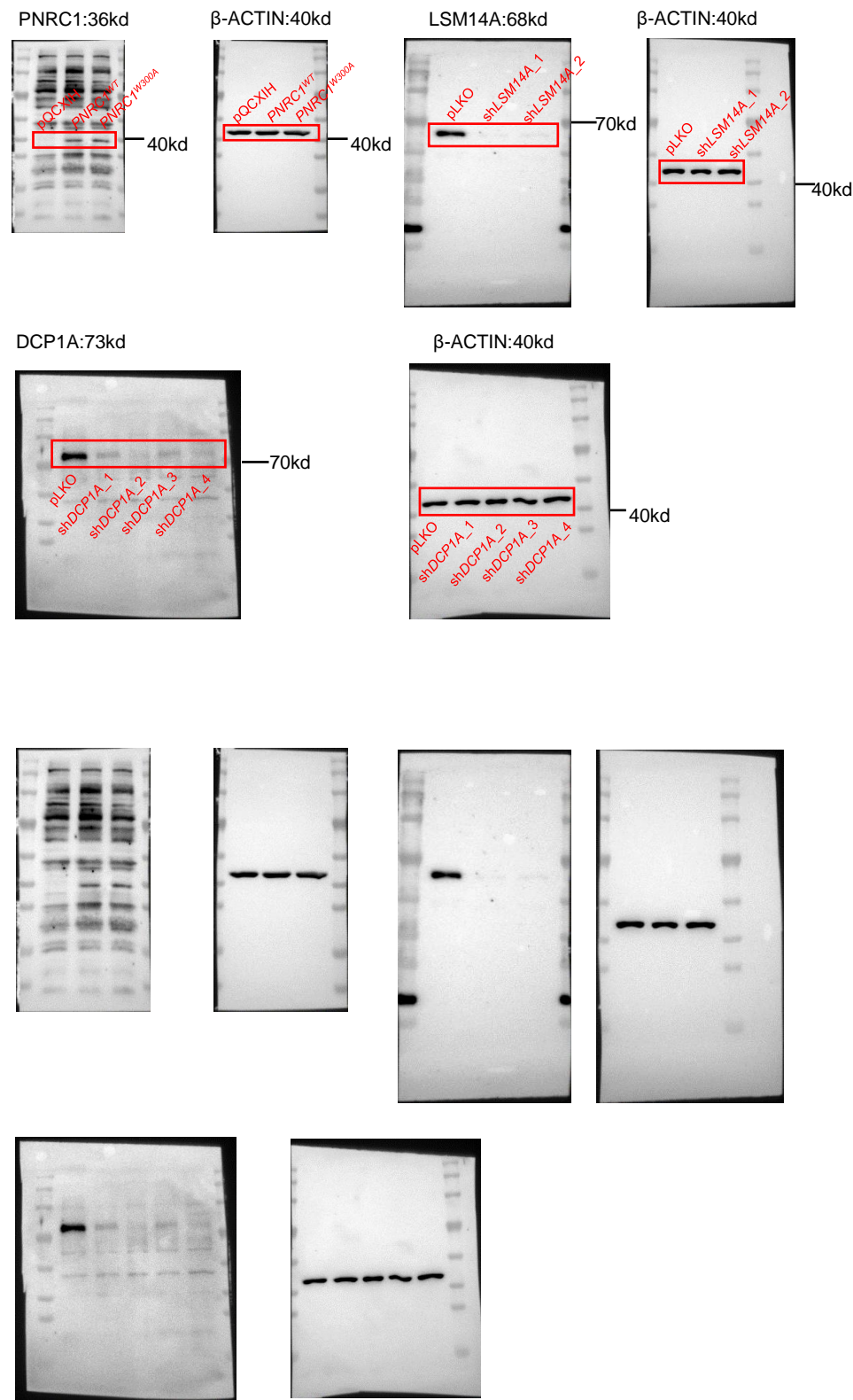

Supplement: Figure 7—figure supplement 5—source data 2. [file elife-88573-fig7-figsupp5-data2.pdf]
